# Microvascular Anastomoses and Beyond: Exploring the Role of Robotic Systems in Microneurosurgery

**DOI:** 10.1007/s11701-026-03427-0

**Published:** 2026-04-25

**Authors:** Giuseppe Esposito, Jennifer Ashley Watson, Martina Giordano, Clarissa A. E. Gelmi, Luca Regli, Pietro Giovanoli, Nicole Lindenblatt

**Affiliations:** 1 Department of Neurosurgery, Clinical Neuroscience Center, University Hospital Zurich, University of Zurich, Zurich, Switzerland; 2 Zurich Microsurgery Lab, University Hospital Zurich, University of Zurich, Zurich, Switzerland; 3 Department of Plastic Surgery and Hand Surgery, University Hospital Zurich, University of Zurich, Raemistrasse 100, 8091 Zurich, Switzerland; 4 Department of Biomedical and Neuromotor Sciences (DIBINEM), University of Bologna - Alma Mater Studiorum, Bologna, Italy; 5 Faculty of Medicine, University of Zurich, Zurich, Switzerland

**Keywords:** Robotic platforms, Microneurosurgery, Microvascular anastomoses, Extracranial-intracranial bypasses.

## Abstract

Robotic assistance has evolved from an experimental concept to an established platform across several surgical disciplines. In microsurgery, dedicated robotic systems have demonstrated feasibility, precision, and reproducibility in performing microvascular anastomoses, nerve coaptations, and lymphatic procedures in reconstructive surgery. In contrast, the adoption of robotics in cranial neurosurgery remains limited, with current clinical applications largely restricted to stereotactic, frame-based procedures such as Deep Brain Stimulation and Stereo-electroencephalography, rather than active microsurgical manipulation of brain, vessels and nerves intracranially. Microsurgical robotic platforms provide technical features including motion scaling, tremor filtration, wristed micro-instrumentation, and improved ergonomics, which closely align with the demands of neurosurgical tasks requiring sustained submillimetric precision on intracranial very small structures even in constrained or eloquent anatomical regions. In this mini-review, we integrate established evidence from robotic-assisted microsurgery to explore the translational potential of these systems in cranial microneurosurgery. Particular attention is given to intracranial microvascular anastomoses as a clinically appropriate entry point, due to the characteristics of the extracranial–intracranial bypass procedures. Also, the dissection of superficial tumors and their arachnoidal planes is considered a potential application, where precision and surgeon endurance outweigh operative speed. Existing evidence remains largely preclinical or exploratory, and robust clinical data are scarce. Further development of neurosurgery-specific instrumentation, optimized workflow integration, and dedicated preclinical and clinical studies are required to define the safety, indications, and potential benefits of robotics in microneurosurgery.

## Introduction

Robotic assistance in surgery has evolved from a conceptual innovation to a reliable platform across multiple surgical disciplines. In microsurgery, robotic systems have demonstrated precision, safety, and reproducibility in microvascular anastomoses in reconstructive and lymphatic surgery [1, 2]. In contrast, their integration into cranial neurosurgery remains limited. Current applications are restricted to stereotactic, frame-based procedures such as Deep Brain Stimulation (DBS), Stereo-electroencephalography (SEEG) and biopsies.

The technical features of microsurgical robotic platforms, such as motion scaling, tremor filtration, and improved ergonomics, suggest potential applicability to neurosurgical tasks requiring sustained submillimetric precision and operator stability. Among these tasks, intracranial microvascular bypass procedures could represent an appropriate entry-point for microrobotic application. Nevertheless, the extension of robotic assistance to fine dissection of very tiny structures, even in anatomically constrained or functionally eloquent cranial regions, has long been met with skepticism, owing to corridor limitations, instrument bulk, and the lack of neurosurgery-specific design in available platforms [3]. Against this background, the present mini-review re-examines previously reported applications in light of recent technological advances.

## Robotics system in neurosurgery: where we currently stand

In cranial neurosurgery, robotic systems are currently used primarily for stereotactic and navigation-based procedures, where they function as high-precision trajectory guidance tools [4, 5]. In contrast, robotic application to active transcranial microneurosurgical manipulation remains limited, with only scarce reports available. A recent systematic review by Gelmi et al. [6] analyzing robotic-assisted minimally invasive cranial neurosurgery identified several studies, most of which were preclinical or cadaveric, with most clinical applications focused on endoscopic skull base approaches or ventricular procedures rather than true microneurosurgical manipulation. Based on the classification reported by Gelmi et al., robotic systems used in cranial neurosurgery can be grouped into three categories: Trajectory-guidance robotic systems, General-purpose surgical telemanipulators, and dedicated microsurgical robotic platforms [6].

“Trajectory-guidance robotic systems” represent the most mature and widely adopted robotic application in neurosurgery, serving as integral tools in functional workflows by enhancing stereotactic accuracy and reducing operator-dependent variability, without direct involvement in microsurgical manipulation.

“General-purpose surgical telemanipulators”, most notably the da Vinci system, have been adapted for selected cranial applications, particularly skull base and ventricular procedures, using articulated instruments and three-dimensional visualization. However, their adoption in neurosurgery remains constrained by the size of the instruments, the lack of dedicated microsurgical optimization and integration with neuronavigation systems, as well as a significant operating room footprint. Consequently, their current role is largely exploratory, supported mainly by preliminary investigations and early clinical case series.

A third distinct category is represented by “Dedicated microsurgical robotic systems”, such as NeuRobot [7], SmartArm, and Concentric Tube Robotic systems. These have been developed to address intracranial constraints through miniaturization, flexible trajectories, and task-specific design, although their use remains largely preclinical [6]. A separate subcategory is represented by microsurgical telemanipulator platforms, such as the Symani^®^ Surgical System and MUSA. These microrobotic systems are designed specifically for fine microsurgical manipulation and microanastomoses, offering motion scaling and tremor filtration. So far, they have seen minimal application in cranial neurosurgery (NCT07140731), highlighting the gap between microsurgical robotic capability and neurosurgical clinical adoption. Within this landscape, the critical unanswered question is not whether robotics can be applied to cranial neurosurgery, but which microneurosurgical tasks are inherently compatible with the strengths and limitations of currently available microsurgical robotic platforms.

### Microsurgical skills: from microvascular anastomoses to extra-intracranial bypass

Microvascular anastomoses are among the most technically demanding manoeuvres in both neurosurgery and plastic/reconstructive surgery, requiring submillimetric accuracy, delicate tissue handling, prolonged operator stability and sustained focus. These requirements place intrinsic limits on manual performance, particularly in deep or confined operative fields, and provide a clear rationale for technological augmentation. Microsurgical robotic platforms have been developed to specifically address these demands through wristed micro-instrumentation and 3D visualization, enabling controlled and repeatable suturing movements under high magnification [1].

Within this conceptual framework, robotic microvascular anastomoses has emerged as the most mature and consistently reported application of microsurgical robotics. Table 1 provides a focused synthesis of the most commonly reported robotic applications in microsurgical practice, derived from representative clinical studies indexed in PubMed (MEDLINE) between January 2010 and December 2025, obtained from a targeted literature search, which was conducted combining the following keywords: “robotic” AND (“microneurosurgery” OR “neurosurgery” OR “microsurgery”). Studies were screened based on predefined inclusion criteria: clinical or translational application of robotic systems in microsurgical procedures, relevance to cranial or peripheral microsurgical techniques, and availability of sufficient technical or outcome-related data. Non-English articles, purely experimental studies without translational implications, and reports lacking procedural detail were excluded. This work is intended to contextualize the established use of robotic platforms in microsurgical procedures and to explore the translational potential of these systems in cranial microneurosurgery.

**Table 1:** Summary of clinical series reporting robotic-assisted microsurgical procedures and platforms employed

Author	Year	Number of patients	Numbers of anastomoses/microsurgical procedures	Type of procedure	Robotic system employed
Garcia JC [8]	2012	3	12	Brachial plexus reconstruction using a sural graft	DaVinci
Miyamoto H [9]	2013	6	6	Nerve transfer	DaVinci
van Mulken TJM [10]	2020	8	14	Supermicrosurgical lymphatico-venous anastomosis	MUSA
Lindenblatt N [11]	2022	5	10	Lymphovenous anastomoses, lympho-lymphatic anastomoses	Symani^®^ Surgical System
van Mulken TJM [12]	2022	8	14	Supermicrosurgical lymphatico-venous anastomosis	MUSA
Grünherz L [13]	2023	1	1	Lymphovenous anastomoses	Symani^®^ Surgical System
Weinzierl A [14]	2023	8	N/A	Lympho-venous anastomosis (LVA), vascularized lymph node transfer (VLNT),	Symani^®^ Surgical System
Grünherz L [15]	2024	4	5	Lymphovenous anastomosis	Symani^®^ Surgical System
Reilly F [16]	2024	12	16	Lymphovenous anastomosis	MUSA
Struebing F [17]	2024	50	94	Venous and arterial anastomoses, nerve coaptations, lymphovenous anastomoses	Symani^®^ Surgical System
Struebing F [2]	2024	16	30	Venous and arterial anastomoses, nerve coaptations	Symani^®^ Surgical System
Von Reibnitz D [18]	2024	67	100	Lymphatic tissue transfer, lymphovenous anastomoses, lympholymphatic anastomoses	Symani^®^ Surgical System
Von Reibnitz D [19]	2024	/	117	End-to-end anastomoses of 2 mm PCA vessel	Symani^®^ Surgical System
Weinzierl A [20]	2024	5	6	Lymphovenous anastomoses	Symani^®^ Surgical System
Imholz C [24]	2025	1	2	Lymphovenous anastomoses	Symani^®^ Surgical System
Watson JA [1]	2025	6		Free flap reconstruction of the scalp	Symani^®^ Surgical System

As shown by Table 1, beyond vascular suturing, robotic assistance has also been applied to nerve surgery and lymphatic reconstruction, supporting its feasibility in complex microsurgical environments [11, 13–15, 20, 22–24]. In particular, robotic systems were relevant for precise nerve coaptation in deep or anatomically constrained fields, such as brachial plexus surgery, where conventional ergonomics and instrument manoeuvrability may be limiting [8, 9]. Importantly, published clinical series consistently indicate that robotic-assisted microanastomoses can be performed safely, with patency rates comparable to conventional microsurgical techniques, up to 100%, and without an increase in procedure-related complications such as flap loss [1, 2]. Although operative times may initially be longer during early adoption phases, subsequent experience demonstrates progressive time reduction, underscoring the relevance of structured training and workflow familiarization [1, 2]. In line with these clinical observations, preclinical motion-analysis studies suggest that robotic microsuturing may achieve greater geometric consistency and reduced variability compared with hand-sewn sutures, potentially reflecting decreased operator fatigue and improved movement stability [21].

**Fig. 1:**
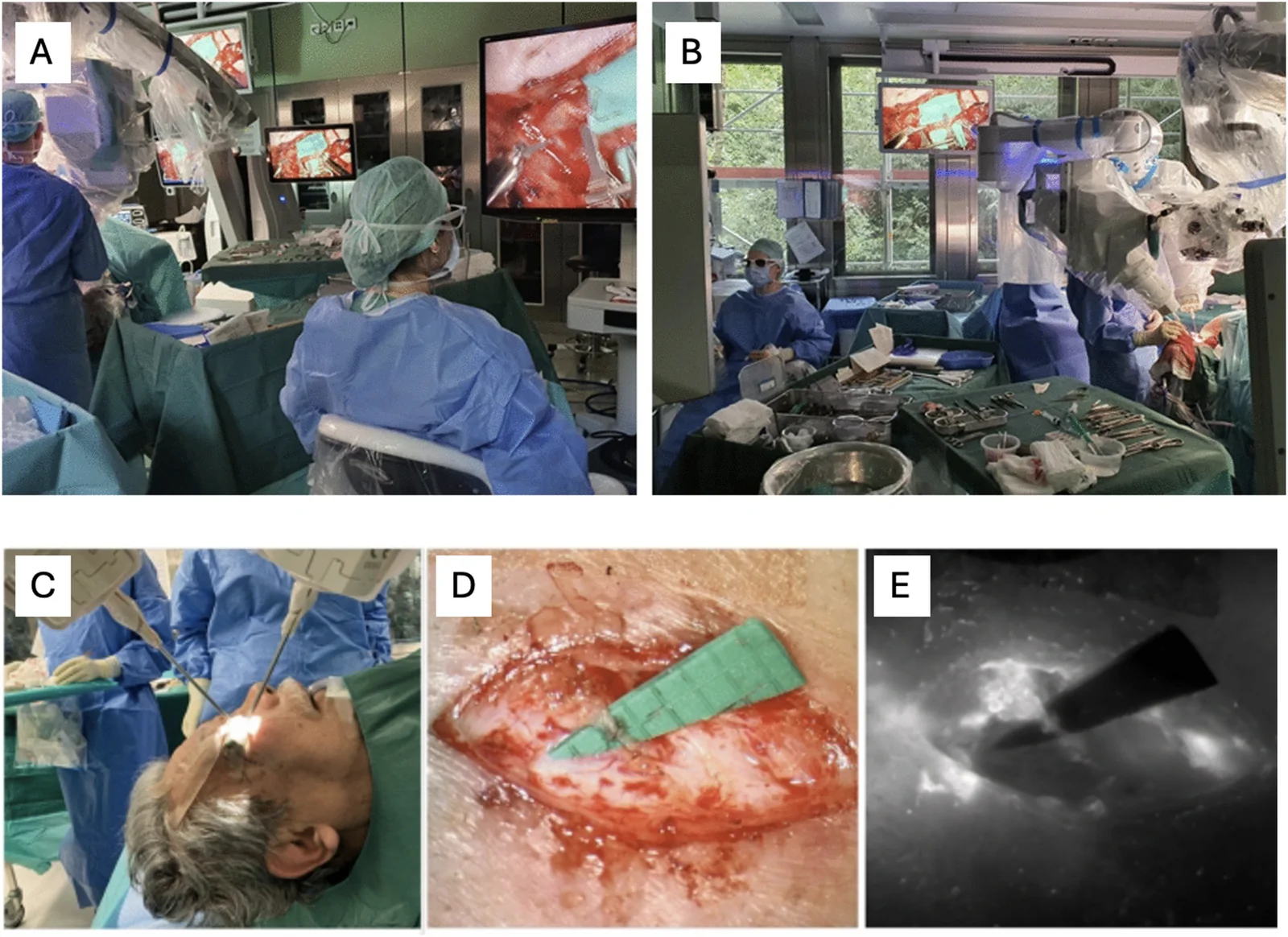
Example of operating room settings and intraoperative view in robotic reconstructive surgery. [1] A, 1B. Reproduced from Watson et al.1 and created under a Creative Commons Attribution 4.0 International License (http://creativecommons.org/licenses/by/4.0/). The Symani^®^ Surgical System is used with an exoscope for visualization. A: The surgeons use the Joysticks to direct the robotic arms without the necessity standing next to the surgical field. B: Via monitor the entire operating team can follow the surgery. If assistance was needed this could be easily provided by an assisting surgeon from the side of the operating table opposite to the Symani^®^ Surgical System.1 C, 1D, 1E. Reproduced from Imholz et al. [24] and created under a Creative Commons Attribution 4.0 International License (http://creativecommons.org/licenses/by/4.0/) C: Intraoperative setup of the Symani Surgical System^®^. D: A robotic-assisted LVA of a 0.2 mm lymphatic vessel was performed in the lower eyelid on each side of the face. E: ICG-angiography showing anastomotic patency

We already reported on an (extra) cranial setup for performing scalp microvascular anastomoses. Figure 1 provides a schematic illustration of a typical operating room configuration for robotic microsurgery, using the Symani^®^ Surgical System, highlighting key aspects of system positioning, surgeon ergonomics, and visualization strategy. Such considerations are particularly relevant when extrapolating robotic microsurgical techniques to neurosurgical environments, where operating room space, instrument crowding, and compatibility with existing equipment represent critical constraints.

Collectively, these findings identify microvascular anastomosis as a potential entry point for robotic assistance in microneurosurgery. This includes STA–MCA bypass, performed for either flow augmentation or flow preservation [25, 26]. This bypass involves end-to-side microanastomoses between a donor scalp artery (the STA) and a cortical branch of the MCA (M4-segment) [27]. The procedure is technically demanding due to small vessel caliber, limited operative space, and delicate neural structures involved [28]. Robotic assistance may enhance submillimetric precision, reduce tissue trauma, and mitigate surgeon fatigue through motion scaling and tremor filtration. Clinical evidence is currently limited, and ongoing studies, including the Symani RESTORE trial (NCT07140731), are evaluating the safety and feasibility of robotic-assisted bypass surgery.

In this context, the neurosurgeon adopts an operative setup analogous to extracranial robotic microanastomoses, operating from a console with dual joysticks and stereoscopic 3D visualization provided by an exoscope. The robotic arms perform the procedure at the bedside, while an assistant supervises the field and manages intraoperative needs. This configuration represents a paradigm shift, with the surgeon interacting with the surgical field through a remote interface rather than operating directly on the patient.

### From microsuturing to microdissection: robotics for tumor resection

Tumor dissection near critical neurovascular structures or within eloquent regions requires exceptional precision and sustained operator performance. In this context, robotic assistance may enhance accuracy, reduce surgeon fatigue during prolonged procedures, and facilitate instrument triangulation within deep or constrained operative corridors, including retrosigmoid, transnasal, and supraorbital approaches.

In other surgical specialties, robotic systems have become standard-of-care by improving maneuverability and precision in anatomically constrained settings, including urology, gynecology, and head and neck surgery [29–31]. Similar benefits have also been reported in hepatobiliary and pancreatic procedures involving tumors adjacent to critical vascular structures [32, 33].

In cranial neurosurgery, a similar ‘space-creation’ principle may help mitigate corridor-related constraints. Analogous to CO₂ insufflation in laparoscopy [34], cerebrospinal fluid release through basal cistern opening promotes brain relaxation and creates a functional operative space for robotic telemanipulation. Coupling this strategy with dedicated microsurgical robotic platforms may reduce corridor-related and instrument–tissue interaction limitations that have historically constrained robotic applications in cranial neuro-oncology and skull base surgery [3].

With specific regard to microsurgical tumor dissection, robotic assistance has been explored primarily outside the cranial cavity. Tigan et al. [35] reported the use of robotic platforms for intraneural dissection and debulking of peripheral nerve tumors, demonstrating reduced incision size and improved surgical precision in anatomically constrained settings.

In cranial neurosurgery, early robotic applications in neuro-oncology have largely focused on deep-seated or intraparenchymal targets. The SmartArm [36] robotic system has been applied to intraparenchymal brain tumor surgery in a pre-clinical setting, including glioma resection, with reported advantages such as enhanced precision within narrow surgical corridors, virtual definition of restricted “no-go” zones, and improved surgeon ergonomics [37]. However, subsequent critical analyses, most notably by Marcus et al. [3], have emphasized that these systems remain constrained by limited workspace, instrument bulk, and the lack of true microsurgical dexterity, particularly when applied to skull base neuro-oncological procedures requiring fine neurovascular dissection. As a result, robotic applications in the deep skull base have remained largely experimental, confined to feasibility reports rather than standardized clinical adoption.

In contrast, a distinct translational rationale emerges when robotic assistance is applied to fine, surface-level microsurgical tasks rather than deep corridor access. Dedicated microsurgical robotic platforms, such as the Symani^®^ Surgical System, are particularly suited to enhance ergonomics and precision during microvascular work performed under high magnification.

This approach may hold significant potential for tumor dissection, provided that appropriate microsurgical instrumentation becomes available. Currently, the Symani ^®^ is limited to microscissors, a needle holder, and microforceps, tools that are ideal for microanastomotic techniques but insufficient for the broader demands of oncological microsurgery. The addition of suction, bipolar coagulation, and ultrasonic aspiration tools would constitute a substantial step forward in enabling the robotic system to be applied to microsurgical resection of brain tumors. Taken together, these experiences suggest that, building on the demonstrated feasibility of robotic integration in microsurgery, selected applications in Neurosurgical Oncology may benefit from robotic assistance. Preclinical validation and clinical studies are required to define indications, safety, and potential advantages in tumour resection near eloquent neural structures.

### Learning curve and surgical confidence

Proficiency in performing microvascular anastomoses using the robotic systems was demonstrated to significantly improve with practice, accompanied by a reduction in operative time. Although overall performance and efficiency correlate with the surgeon’s prior years of experience, a distinct learning curve is evident across all levels of training, including medical students, residents, and attending physicians [16]. Similarly, the systematic review by Cannizzaro et al. suggests that while robotic procedures initially take longer, time efficiency improves significantly with experience [38]. With surgeons becoming progressively more confident in the use of robotic tools, a translation of these skills to more nuanced applications can be expected, such as the precise disconnection of the tumour capsule from cranial nerves or microcoagulation of small feeders. These are actions where millimetric control outweighs speed, and where robotics can offer measurable safety advantages.

## Discussion

Robotics application in microneurosurgery has so far been limited. However, evidence from microsurgical disciplines suggests that robotic assistance may apply to microneurosurgical tasks. A recent clinical case series by Eroglu et al. [39] represents the first report of neurosurgical procedures using a robotic telemanipulator (the da Vinci platform), demonstrating the feasibility of robotic integration not only in the skullbase procedures [40], such as trans-oral approach, but also in arachnoid cyst fenestration, and peripheral nerve surgery.

Moving beyond telemanipulation, preclinical evidence [41] has demonstrated the feasibility of robotic-assisted microsuturing and microvascular anastomoses, while early clinical experiences are beginning to support their translational potential1.

Within this framework, microvascular anastomoses emerge as a pragmatic entry point for robotic integration, in particular for STA–MCA bypass, the most common neurosurgical bypass procedure. In this operation, an extracranial donor artery (the superficial temporal artery - STA) and an intracranial recipient artery (the middle cerebral artery - MCA) are connected via an end-to-side microanastomosis. The blood flow is therefore rerouted from extra- to intracranially. In STA-MCA bypass procedures, the recipient artery is typically a superficial cortical branch of the MCA (a M4-segment) [1] Hence, the microanastomosis is performed on the brain surface and the cortical recipient artery tolerates well the temporary occlusion (normally made under 10–20% blood pressure increasement). The potential increasement in operative time at the beginning can be therefore well tolerated. Thereafter, operative time is expected to decrease as experience increases, as reported in other disciplines.1 In high-flow bypass the situation is different: these are procedures performed in the depth, with a proximal and deeper recipient. This deep bypass recipient could not tolerate the temporary occlusion in the same way of a cortical superficial one. This means that only surgeons whose learning curve and skills have been consolidated by performing STA-MCA bypasses could consider performing high flow bypass.

Tumor dissection represents a second, more exploratory domain of application. While current robotic instrumentation is well-suited for suturing tasks, the lack of essential tools still limits its role in intracranial oncological microsurgery. Future technological developments in instrument integration will be crucial to enable broader application, particularly for fine arachnoid dissection or tumor debulking.

Given the high precision requirements inherent to neurosurgery, robotic systems might offer a structured means of enhancing surgical accuracy while improving ergonomics. Robotic microsurgery does not replace manual microsurgical expertise and remains dependent on the skills of an experienced microsurgeon. Rather, it functions as an adjunct to conventional techniques. Successful implementation requires dedicated training in both traditional microsurgery and robotic workflows, as well as the ability to manage intraoperative challenges, including technical limitations or system-related issues.

Despite differences in surgical setting and physiological context between EC–IC bypass and lymphatic supermicrosurgery, the robotic execution of the anastomotic task may be, at least in part, transferable across domains, considering the enhanced precision, motion scaling, and tremor filtration. Therefore, robotic assistance may enable highly precise and controlled stitch placement, potentially minimizing vessel trauma and contributing to reliable anastomotic patency.

### Toward a robotical neurosurgical workflow

Integration of robotic platforms into neurosurgical practice would also require adaptations of the operating room (OR) workflow.

First, robot positioning and docking would need to be incorporated into surgical planning, with predefined trajectories and arm configurations established before incision to avoid interference with the microscope, neuronavigation, and anesthesiology equipment. This may shift part of surgical preparation to a preoperative setup phase, similar to current workflows in robotic pelvic or head and neck surgery.

Second, visualization workflows would likely evolve. Robotic microsurgery relies on teleoperation and three-dimensional visualization, often via digital exoscopes or integrated camera systems, which may partially replace conventional microscope use or require hybrid setups. This would necessitate coordinated screen placement, surgeon console positioning, and team communication protocols to maintain shared situational awareness.

Third, instrument exchange and assistance roles would be modified. Robotic systems allow the surgeon to control multiple instruments independently, potentially reducing the need for continuous manual assistance during microsurgical steps. As a result, the role of the assistant may shift toward system monitoring, suction management, and troubleshooting rather than direct instrument handling.

Finally, training and safety protocols would need to be adapted. OR teams would require familiarity with robotic setup, troubleshooting, and emergency undocking procedures to ensure rapid conversion to conventional microsurgery in the event of technical failure, preserving patient safety. Meaningful integration of robotic systems into neurosurgery would require the development of procedure-specific instrumentation. Using the Symani^®^ Surgical System as an example, currently available nano-wristed instruments include scissors, forceps, and vessel dilators. However, broader neurosurgical applications would necessitate the integration of additional tools, such as microdissectors, bipolar coagulation, suction–aspiration devices, and potentially ultrasonic aspiration systems, intraoperative monitoring system (direct cortical or subcortical stimulation) to address the specific requirements of intracranial surgery.

### Limitations of robotic microsurgery in neurosurgical applications

The limitations of robotic platforms in neuromicrosurgery have been previously described [6, 40]. The steric footprint and physical presence of robotic arms, together with their fixed angles of approach, may interfere with the narrow working corridors typical of microneurosurgery, representing a relevant technical limitation for cranial applications. In addition, the lack of haptic feedback remains a significant challenge, only partially compensated by visual cues and progressive adaptation along the learning curve, and warrants further technological development. Cost-related factors, including acquisition, maintenance, and training, are substantial, although partially mitigable through shared-use models across surgical specialties. Finally, the applicability of robotic systems in neurosurgery should be interpreted in light of the learning curve, with operative times decreasing as experience increases. In parallel, enhanced precision, motion scaling, and tremor filtration may offer a potential surgical advantage.

## Conclusion

Building on the established clinical performance of robotic microsurgery in reconstructive, lymphatic, and nerve procedures, robotic assistance shows a sound translational potential for selected microneurosurgical applications. Core platform features such as motion scaling, tremor filtration, wristed micro-instrumentation, and ergonomic telemanipulation directly address key technical limitations of manual microneurosurgery in confined operative fields. With the currently available instrumentation, microvascular procedures may represent one of the most immediate and clinically relevant areas of application, with extracranial–intracranial bypass surgery, particularly STA–MCA anastomoses, and arachnoidal tumor dissection potentially serving as initial proof-of-concept scenarios for robotic integration. Robotic platforms should be viewed as precision-enhancing adjuncts rather than replacements for surgical expertise, with further progress depending on neurosurgery-specific instrumentation, workflow integration, and structured preclinical and clinical validation.

## Data Availability

No datasets were generated or analysed during the current study.
